# Determination of collagen content within picrosirius red stained paraffin-embedded tissue sections using fluorescence microscopy

**DOI:** 10.1016/j.mex.2015.02.007

**Published:** 2015-02-21

**Authors:** Benjamin Vogel, Hanna Siebert, Ulrich Hofmann, Stefan Frantz

**Affiliations:** a Comprehensive Heart Failure Center (CHCF), Universitätsklinikum Würzburg, Germany; b Medizinische Klinik und Poliklinik I, Universitätsklinikum Würzburg, Germany; c Universitätsklinik und Poliklinik für Innere Medizin III, Universitätsklinikum Halle (Saale), Halle (Saale), Germany

**Keywords:** Collagen determination in tissue sections by PSR fluorescence, Picrosirius red (PSR) fluorescence, Collagen determination, Planimetry, Myocardial infarction, Digital image processing

## Abstract

Picrosirius red (PSR) staining is a commonly used histological technique to visualize collagen in paraffin-embedded tissue sections. PSR stained collagen appears red in light microscopy. However it is largely unknown that PSR stained collagen also shows a red fluorescence, whereas live cells have a distinct green autofluorescence. Both emission patterns can be detected using standard filter sets as found in conventional fluorescence microscopes. Here we used digital image addition and subtraction to determine the relative area of the pure collagen and live cell content in heart tissue in a semi-automated process using standard software. This procedure, which considers empty spaces (holes) within the section, can be easily adapted to quantify the collagen and live cell areas in healthy or fibrotic tissues as aorta, lung, kidney or liver by semi-automated planimetry exemplified herein for infarcted heart tissue obtained from the mouse myocardial infarction model.

• Use of conventional PSR stained paraffin-embedded tissue sections for fluorescence analysis.
• PSR and autofluorescence images are used to calculate area of collagen and area of live cells in the tissue; empty spaces (holes) in tissue are considered.
• High throughput analysis of collagen and live cell content in tissue for statistical purposes.

## Graphical abstract

**Figure:**
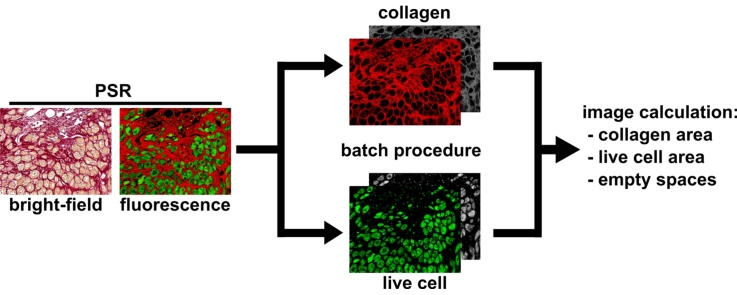

## Method details

Picrosirius-red (PSR) is a well-known method to stain collagen in histology. However it is rather unknown that PSR stained tissue sections can also be analyzed brilliantly by fluorescence. Here we provide an easy and fast procedure how to determine collagen and live cell content for descriptive or statistical purposes from PSR stained tissue sections using fluorescence microscopy, semi-automated image processing and standard software.

## Required equipment and software

- PSR stained paraffin-embedded tissue section (here: heart) [5].
- Epifluorescence microscope (here: Zeiss Z1m Imager) with standard filter sets capable to detect FITC and Rhodamine (here: “FITC”: Ex: 450–490 nm, Em: 500–550 nm, “Rhodamine”: Ex: 538–562 nm, Em: 570–640 nm).
- Fluorescence camera with appropriate acquisition software (here: Axiocam MRm with Axiovison 4.8) capable of producing 8-bit images [jpg or a proprietary (batch) convertible file format]. A confocal microscope can be used as well as described [1], but is not necessary.
- Zeiss ZEN lite 2011 SP1 1.0.1.0 (freeware, Zeiss, Oberkochen, for batch extraction of *.jpg channels from *.zvi files).
- Adobe Photoshop 7.0 or newer (for batch image downscaling from color to 8-bit grayscale images, image calculation and processing).
- DirPrintOK (freeware, for file name inclusion to MS Excel, www.softwareok.de).
- MS Excel 2010 or similar program (for data acquisition and calculation).

The method here is described for the use with a Zeiss Z1m Imager epifluorescence microscope, which results in the acquisition of a proprietary *.zvi file which contains the actual fluorescence images channels and metadata. However any other fluorescence microscope can be used if images can be acquired or finally converted to individual 8-bit jpg channel files. It is noteworthy that freeware software as ImageJ or CellProfiler instead of Abode Photoshop might also be capable of performing the procedures presented here.

## Image acquisition

1. Carefully choose the field of view according to your experimental design. Avoid field of views with artefacts due to tissue cutting or incorrect attachment of the section to the slide. Always use the same magnification for all image acquisitions (recommended: 20× objective).
2. Set up exposure times for autofluorescence (green) and collagen (red) channels. Do not acquire other channels than the red and green fluorescence channel since this will increase data amount and delay the following processes. Use a range indicator in your acquisition software. Do not overexpose green and red fluorescence channels. For correct determination of collagen content make sure the image is focused to the (red) collagen channel, especially tissue sections >7 μm. Focusing to the (green) autofluorescence channel is not recommended. The green channel seems to be out of focus from time to time compared to the collagen scaffold probably due to the fixation, embedding and cutting procedure.
3. After setting the exposure time for all channels individually, acquire the two channels and save as a proprietary Zeiss *.zvi file. Non-Zeiss microscopy users should make sure that they can (batch) extract the individual channels as an 8-bit jpeg-file in highest possible quality from their proprietary file format. Otherwise each image channel should be saved separately after acquisition in the highest possible quality to a *.jpg file. The separate channels should be named uniquely for each field of view and marked clearly after the filename (filename_c1 or _c2.jpg).
4. Proceed with another field of view and the acquisition of individual channels as described. Each field of view can be exposed and focused individually to fulfil the mentioned criteria.

The amount of files created during image acquisition can easily lead to hundred files or more for the statistical analysis of an experiment. However handling of this large amount of files is no problem due to the batch processes described within this procedure here. All acquired *.zvi files to be analyzed should be located in one folder to speed up subsequent processes.

## Batch image extraction from *.zvi to *.jpg or *.tif channel files

This step is used to batch extract the individual channel files (collagen and live cell) as *.jpg or *.tif from the proprietary *.zvi file format.

Very important information regarding the selection of *.jpg (default selection) or *.tif file format for further analysis

Generated channel files (*.tif or *.jpg) do not have to be stored permanently after analysis, since they can be easily re-extracted from the original *.zvi files. Uncompressed *.tif files are of better quality, however also much larger than highest quality *.jpg files, which might be relevant if older computers are used. In a trial where *.jpg and *.tif channel files were directly compared regarding the generated final values, no statistically significant differences could be detected between file formats. However quality loss in *.jpg files might be relevant, if subtle differences are to be hest possible sensitivity the use of the *.tif file format is recommended.

Video: “Vogel et al ZEN batch.mp4”.

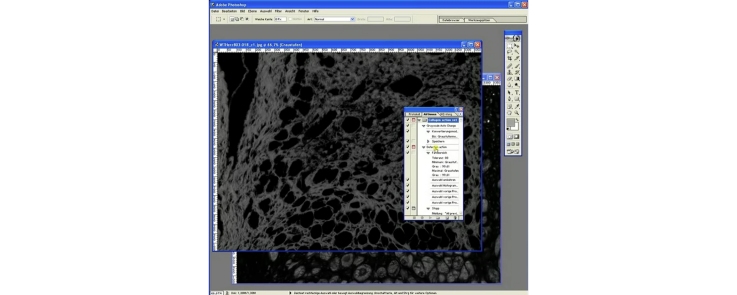

1. Start ZEN lite 2011 SP1 software (version 1.0.1.0; prior versions have problems which batch processing).
2. Select the ribbon “Processing” > “Batch”. Batch method: “Single file export”.
3. Check the “Use Input Folder as Output Folder” box or choose another folder (Browse) before adding the files to the batch.
4. Add the all *.zvi files to the batch extracted by clicking the “+ Add…” button.
5. Mark the first file.
6. Method parameters will now be active: Filetype: jpg (default selection, Quality: 100%) or tif (highest quality files; Check conversion to 8 bit; Compression: None); Resize 100%; Check boxes “Apply Display Curve and Channel Color” and “Individual Channels”. All other boxes must be unchecked (especially “create folder”).
7. Press “Copy Parameters”, mark the second file, then “Ctrl” and “A” on your keyboard to mark all files in the batch.
8. Press “Paste Parameters”. All parameters selected for the first file in the batch procedure should now be applied to all other files. Check randomly for correct pasting of parameters
9. Press the “Run Batch” button to start batch.

This batch procedure should result in two *.jpg or *.tif files per *.zvi file in your output folder. The red and green channels will automatically receive a _c1 (here: red) and _c2 (here: green) correspondingly added to their original file names for differentiation. These files should be cut and pasted to a new folder (e.g., channels).

## Batch conversion to grayscale

This step is used to convert all the extracted channel jpg files from color. Adobe Photoshop was used since it harbors the most powerful color to grayscale converter tested. However any other converter can also be used if appropriate.

Video “Vogel et al Grayscale batch.mp4”.

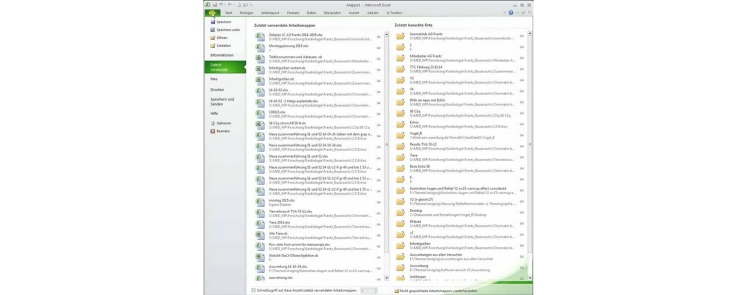

1. Start Adobe Photoshop 7.0.
2. The following step is only necessary once. Load the batch procedure file (check Supplementary data) to generate a custom batch grayscale converter (separate versions for *.jpg ([JPEG] and *.tif [TIFF] files available) and actions for image processing (corresponding Collagen action set JPEG or TIFF wo hint.atn file removes the hint, which occurs during image calculation to inform the user about the events happening; speeds up processing; for experienced users only).

Video “Vogel et al load and modify actions.mp4”.

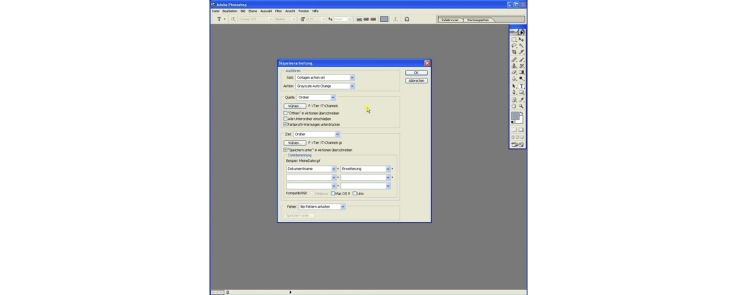

4 Click on “file” > “Automate” > “Batch”.
5 In the “Batch” window use following settings: set: “Collagen action set”, action: “Grayscale Auto Change”. Source: folder; uncheck boxes all boxes but “suppress color profile warnings” and “overwrite save as in actions”.
6 For “source” choose the folder with the extracted channel *.jpg or tif files.
7 For “target” choose a new folder (e.g., “channels gs”).
8 Data naming should be data “filename” + “_gs” (grayscale) + “extension”
9 Start the batch by pressing the “Ok” button. All images are now automatically loaded, grayscale reduced, saved as “filename_c1/c2_gs.jpg or tif” and closed.
10 After batch processing check the target folder. You should locate channel *.jpg or tif files converted to grayscale and renamed with a “_gs” attachment to the end of the original filename. Amount of files should be the same as after extraction of channel files from *.zvi files.

## File name insertion to MS Excel

This step describes the import of file names to Excel for correct assignment of analyzed data without typing, which is especially comfortable when working with a large amount of files. This procedure is especially useful if files are irregularly named or in interrupted sequence, which would normally make manual typing mandatory.

Video: “Vogel et al file name batch.mp4”.

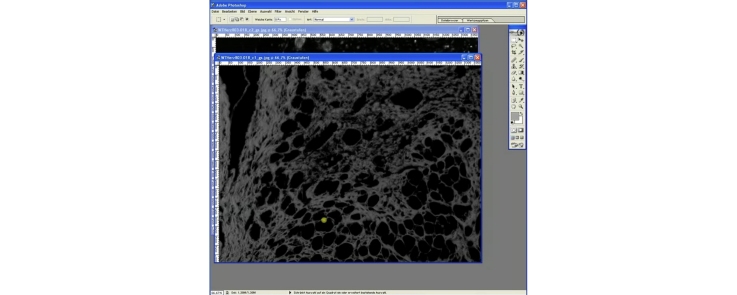

1. Start DirPrintOK (This freeware file explorer allows to save the MS Windows file structure in a Excel-readable *.txt file).
2. Locate the folder with the *.zvi files (not the folders with the extracted *.jpg or tif files or the grayscale converted *._gs.jpg or tif files).
3. Delete all shown attributes but the file name by clicking right on the attribute bar and unchecking. Sort files in the folder alphabetically or reverse by clicking on “name”.
4. Click file > export. Save the file, which includes the file structure in *.txt format.
5. Open the file and delete all lines which do not contain the needed file names. Save the file.
6. Start MS Excel (here: version 2010).
7. Click on data > from text
8. Open modified *.txt file, pass the first window by clicking “next”. In the next window you can choose any separators like “. “ or “ | ” e.g., to move file extensions from file name into a separate column (“example.zvi” > “example” and “zvi”).
9. Click on “make ready” and confirm to get back to your sheet.
10. Delete any columns with unwanted information so that only the file names remain.
11. Include columns with headings e.g., “total area without empty spaces”, “collagen area” and “live cells area”.
12. For overview purposes prepare calculations in cells for relative area of collagen and live cells in the field of view [=relative area of collagen or live cells (pixels)/total area without empty spaces(pixels) × 100].

## Image calculation to determine absolute tissue, collagen and live cell area in the field of view

In this step the two corresponding gray scale reduced channels (filename_c1/c2_gs.jpg or tif) are used for image calculation and area determination in pixels. By image addition of both channels (collagen image + live cell image) the whole section area without empty spaces is achieved. Live cell area (live cell image–collagen image) and collagen area (collagen image–live cell image) in the section are both established by image subtraction.

Video: “Vogel et al image calculation.mp4”

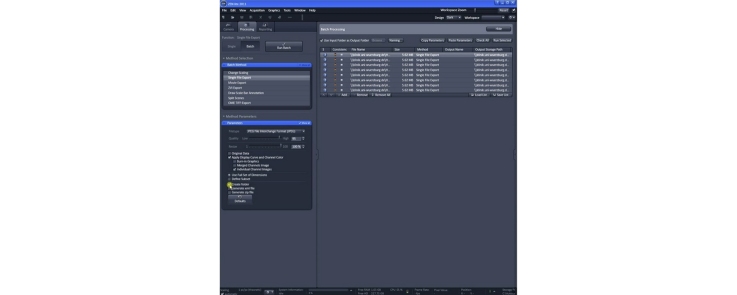

1. Start Adobe Photoshop 7.0 (Important: Step 2 from “Batch conversion to grayscale” must have been already performed. Action file must be loaded.
2. Drag and drop two corresponding channel files (collagen and live cells) converted to grayscale (e.g., filename1_c1/c2_gs.jpg or tif) to the program.
3. Determination of total tissue area without empty spaces (addition):
  a. Highlight the window with the collagen or live tissue channel.
  b. Click on image > image calculations; a new window opens.
  c. The actual highlighted channel is always stated next to “target:”. The not highlighted channel has to be selected in “Source” manually. Set “Mode” to “addition”. Press “Ok” to confirm the settings and perform image calculation.
  d. Check if image calculation makes sense. A merged image of collagen and live tissue should appear.
  e. Press “Shift” + “F5” to start automated area measurement.
  f. The complete tissue area, excluding holes in the section is marked and the histogram window opens automatically. Write down the number next to “pixel:” into your prepared Excel sheet to the corresponding file name and column (total area without empty spaces). Press “Ok” to close the histogram window. Close the following hint by clicking “Ok” (Experienced users: remove this hint by using “Collagen action set JPEG/TIFF wo hint.atn” to speed up the process). All changes and calculations are now reset.
4. Determination of collagen area (subtraction):
  a. Highlight the window with the collagen channel.
  b. Click on image > image calculations; a new window opens.
  c. The actual highlighted channel is always stated at “target:” The non-highlighted channel has to be selected in “Source” manually. Set “Mode” to “subtraction”. Press “Ok” to confirm the settings and perform image calculation.
  d. Check if image calculation makes sense. A subtracted image with only collagen fibers should appear.
  e. Press “Shift” + “F5” to start automated area measurement.
  f. All collagen in the area in the field of view is marked and the histogram window opens automatically. Write down the number next to “pixel:” into your prepared Excel sheet to the corresponding file name and column (collagen area). Press “Ok” to close the histogram window. Close the following hint by clicking “Ok” (Experienced users: remove this hint by using “Collagen action set JPEG/TIFF wo hint.atn” to speed up the process). All changes and calculations are now reset.
5. Determination of live cell area (subtraction):
  a. Highlight the window with the live cell channel.
  b. Click on image > image calculations; a new window opens.
  c. The actual highlighted channel is always stated next to “target:”. The not-highlighted channel has to be selected in “Source”. Set “Mode” to “subtraction”. Press “Ok” to confirm the settings and perform image calculation.
  d. Check if image calculation makes sense. A subtracted image with only live cells should appear.
  e. Press “Shift” + “F5” to start automated area measurement.
  f. All live cells in the area in the field of view are marked and the histogram window opens automatically. Write down the number next to “pixel:” to your prepared Excel sheet to the corresponding file name and column (live cells area). Press “Ok” to close the histogram window. Close the following hint by clicking “Ok” (Experienced users: remove this hint by using “Collagen action set JPEG/TIFF wo hint.atn” to speed up the process). All changes and calculations are now reset.
6. Close both files without saving and repeat all steps for a new set of images until all images are processed.
7. The amount of all pixels in the image (total acquired image area = image size) is determined by clicking on any of the images, press “Crtl” + “A” (mark everything), then click on image > histogram (pixel:). This is necessary only once for each acquisition camera and only e.g., if the “area of empty space or hole” in the tissue is to be determined.

## Adjusting the threshold for detection of collagen, live tissue and empty space area

The automated detection of collagen, live tissue areas and empty space areas is based on a threshold detection method for the grayscale image. In the included action file this threshold (image > replace color > tolerance) is initially set to 88 due to empiric definition in our lab to achieve around 1.0%. collagen content in the healthy heart. All shades of gray lower than this value will not be included in the determined area. However, it might occur that some faint, but anyhow distinct structures might not be detected correctly using this threshold in certain questions. The threshold can be adjusted manually e.g., to 50, but also lower, to prevent this, if necessary. To change the tolerance within the loaded action set perform the following steps

Video “Vogel et al load and modify actions.mp4”:

1. Start Adobe Photoshop 7.0.
2. Load any grayscale image.
3. Click to “Window” > “Actions” to open the “Action” window. Click the “Actions” ribbon. Only one action should be loaded (e.g., Collagen action set.atn). If necessary collapse the set by clicking on the arrow on the left side. Locate the action subset “Detection action”. Double click on “Color range”. A new window should open, where “Tolerance” can be adjusted manually e.g., 50. Press “Ok” after adjustment.
4. Save the changed action set by clicking once on the “Collagen determination action set” to highlight. Then click on the small arrow in the upper left corner. Locate “Save action”. Save to a new file e.g., Collagen action set 50.atn.

A visual comparison between the original gray scale image and the areas automatically marked within the image with tolerance set to 88 (collagen area: 741,961 pixels, 100.0%) and 50 (collagen area: 941,581 pixels, 126.9%) is demonstrated in Fig. 3. Tolerance reduction from 88 to 50 leads to a 27% increase in the detected area in this example. It furthermore has to be stressed that areas and determined values of an image analyzed by this procedure are reproducibly the same when the image is re-analyzed with the same tolerance setting. Therefore the threshold value used for analysis should be mentioned whenever the method is used and most importantly must not be changed during an experiment or during batch processing for proper result comparison and reproducibility.

To underline intra- and inter-assay reproducibility of this procedure the statistic values of four data sets acquired by different researchers, on different dates, in different myocardial infarction experiments are provided and compared in Table 1. In summary the described procedure is a useful tool for collagen determination or general tissue description in different questions, tissues, organisms and animal models using a widely available PSR stained procedure.

## Additional information

Picrosirius red (PSR) staining is a widely used histological technique to visualize the distribution of collagen in healthy and fibrotic tissue sections by bright-field or polarization microscopy [6]. However collagen quantification by polarization microscopy is quite elaborate and therefore not applicable for a rapid analysis of wide views per section, which however is essential for comprehensive analysis of non-homogenously distributed fibrosis in various tissues.

**Fig. 1:**
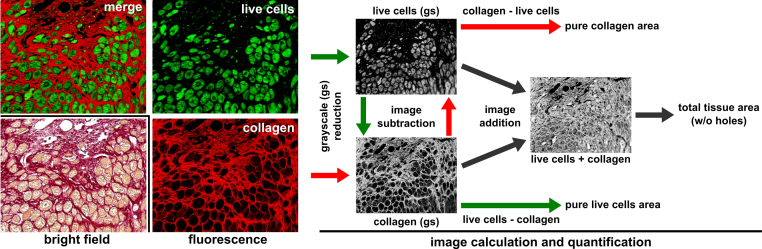
Work-flow for determination of collagen and live cell area using PSR fluorescence. It is rarely known that conventionally PSR stained paraffin sections (light microscopy, bright-field) demonstrate a red collagen and a green (presumably live cell content) auto fluorescence (merge), which can be separated into their distinct individual fluorescence channels. Collagen, live cell content and holes in tissue can be determined from these fluorescence images by image reduction to grayscale (gs) followed by image calculation (addition, subtraction) in a semi-automated batch procedure. Shown images are from the borderzone of an infarcted heart. (For interpretation of the references to color in this figure legend, the reader is referred to the web version of this article.)

It is rather unknown that collagen in PSR stained paraffin-embedded tissue sections can also be visualized brilliantly and with high sensitivity by fluorescence microscopy [1]. This method was originally developed for the three-dimensional visualization of collagen fibers in the heart using confocal microscopy. We observed that the therein described disturbing cellular background fluorescence in heart can only be found presumably in live cells in conventionally stained PSR sections of infarcted hearts (Fig. 1). This is backed up by the fact that cardiomyocytes in tissue and in cell culture demonstrate a strong green autofluorescence due to lipofuscin expression [3], [4]. Interestingly a clear discrimination between extracellular PSR stained red collagen fibers and intracellular green autofluorescent live cells in heart tissue can be found with high confidence and little spectral overlap using conventional filter sets and an epifluorescence microscope.

Based on this observation we describe a digital image processing procedure to determine relative extracellular collagen or live cell tissue content using the (auto) fluorescence properties of conventionally PSR stained paraffin-embedded tissue sections as used commonly in histology. Image calculations as used here have been described previously to reduce unwanted lipofuscin autofluorescence in immunofluorescence [4]. The procedure presented here is easy to apply, uses standard available software and custom semi-automated batch processes to analyze a large amount of field views within a short time. The method allows determining relative collagen and live cell content in a field of view and subtracts empty spaces or holes in the field of view automatically. Therefore the procedure is ideally suited for statistical purposes e.g., comparison of an untreated vs treated cohort after myocardial infarction or pathologic hypertrophy (Fig 1).

**Fig. 2:**
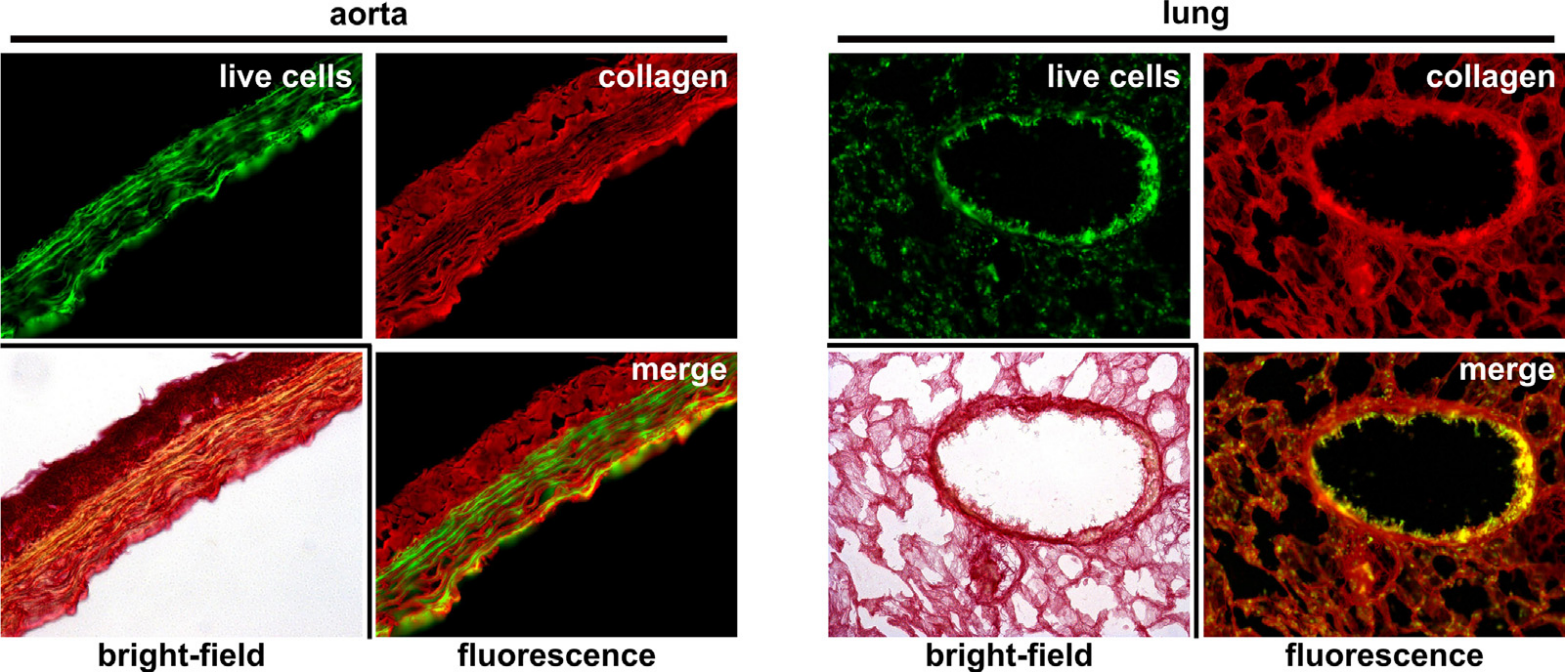
PSR fluorescence can be observed and applied to other tissues than heart. Conventionally PSR stained paraffin sections (light microscopy, bright-field) from aorta (left) and lung (right) also demonstrate specific fluorescence properties (merge) as described in Fig. 1. Red collagen and green (presumable live cell content) auto fluorescence might also be used for quantification of tissue properties using the procedure described here. Other tissues might also be suitable. (For interpretation of the references to color in this figure legend, the reader is referred to the web version of this article.)

**Fig. 3:**
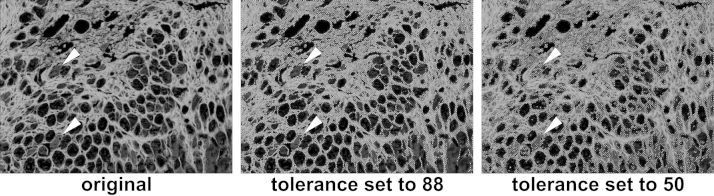
Influence of tolerance setting on the automated area detection. Discrimination between specific areas and holes in tissue during the automated area detection is mediated by a grayscale threshold. A lower threshold detects more structures, which can be useful. However threshold must never be changed within one experiment and should be stated whenever using this procedure. Arrows mark the same positions in the original image and in the areas with threshold set to 88 and 50. Note the distinct changes in the area detection marked by the arrows.

As an example, the procedure is presented for a image depicting the border zone of an infarcted mouse heart 8 weeks after permanent ligation of the left descending coronary artery [2]. The method should be easy to adapt for other tissues where collagen and fibrosis might occur as aorta, lung and perhaps liver and kidney. Analysis of PSR stained lung and aorta tissue from rat indeed reveals a strong red fluorescence corresponding to red stained tissue content by bright-field analysis (Fig 2). Furthermore a discriminable green autofluorescence can be observed in these tissues. However it has to be checked in detail if red fluorescence is indeed collagen and autofluorescence corresponds to live cells as in heart tissue. Quantification of structural differences by autofluorescence subtracted collagen content in these tissues seems possible at least.

## Funding

This work was supported by grant of the German Bundesministerium für Bildung und Forschung (BMBF01 EO1004) (S.F.).

## Conflict of interest

None declared

## Figures and Tables

**Table 1:** Collagen content from non-infarcted heart tissue (septum) from C57BL6/J control animals in different myocardial infarction experiments (group 1–4) were determined independently by different researchers, at different dates. Field of views containing large vessels are generally omitted from image acquisition in this question to rule out falsification of septal collagen content. Note that the groups are statistically not different (*P*-value), and collagen content is stable intra-assay (compare minimum, maximum within groups) and inter-assay (compare mean, SD between groups).

	Group 1	Group 2	Group 3	Group 4
Number of values (*n*)	15	14	13	8
Minimum	0.06	0.09	0.03	0.06
Maximum	1.93	1.94	1.67	1.67
Mean	0.39	0.66	0.50	0.73
Std. deviation (SD)	0.45	0.52	0.58	0.64
*P*-value (one-way ANOVA, Tukey)	>0.05	>0.05	>0.05	>0.05
