# Supplementary figures and images for: Determination of collagen content within picrosirius red stained paraffin-embedded tissue sections using fluorescence microscopy
Source: MethodsX. 2015 Feb 21;2:124–34. doi: 10.1016/j.mex.2015.02.007 (PMC4487704; doi:10.1016/j.mex.2015.02.007)

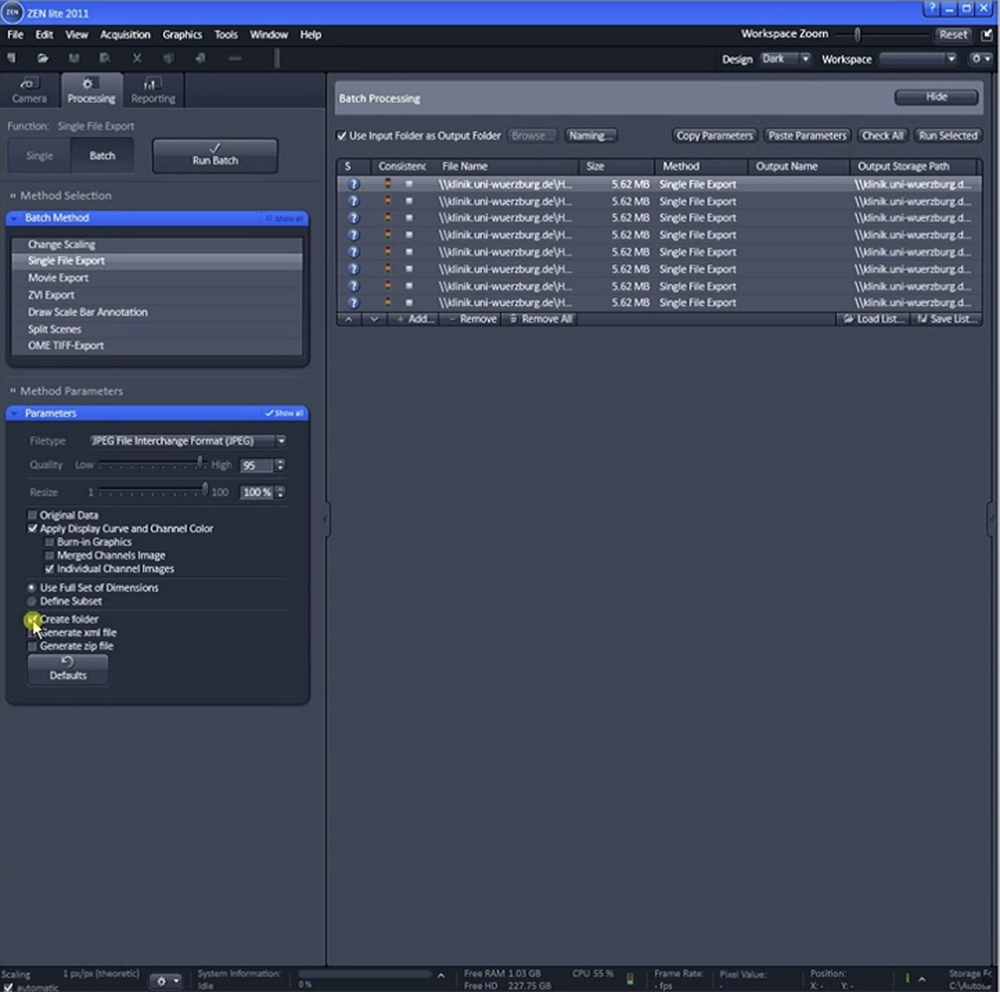

Supplement: Supplementary file 1 [file mmc9.jpg]

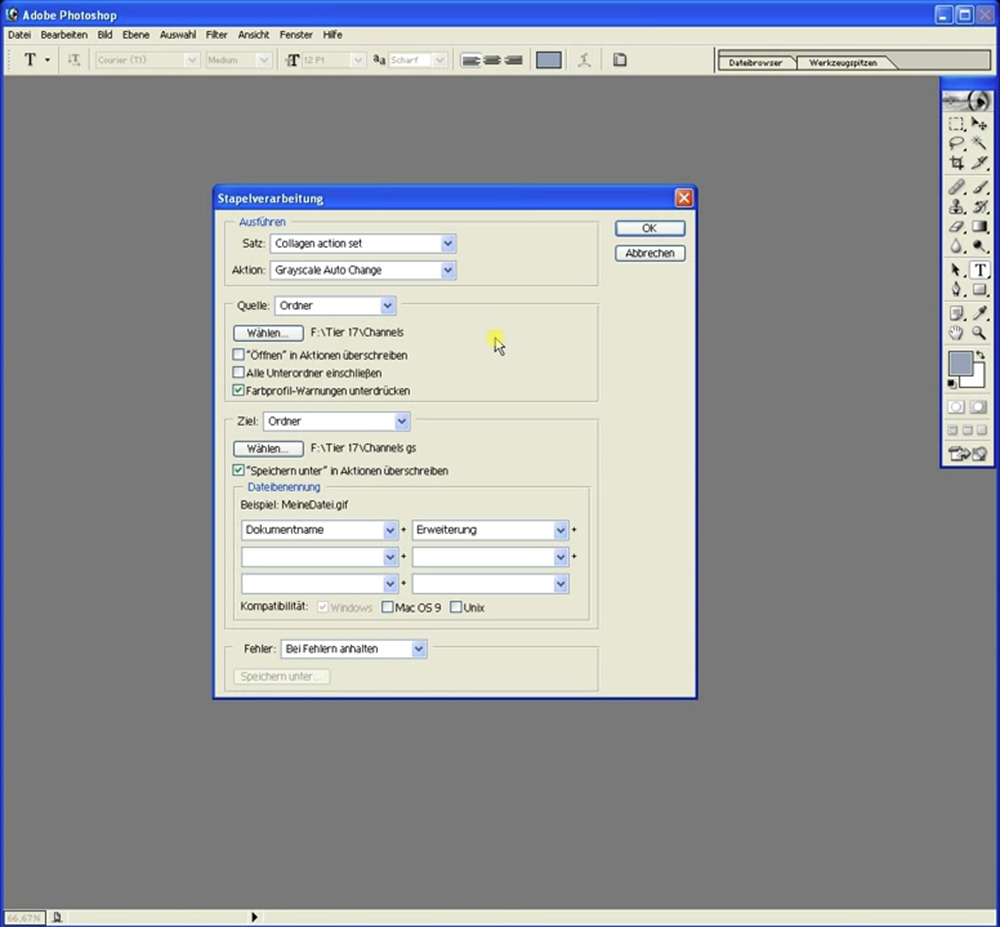

Supplement: Supplementary file 2 [file mmc7.jpg]

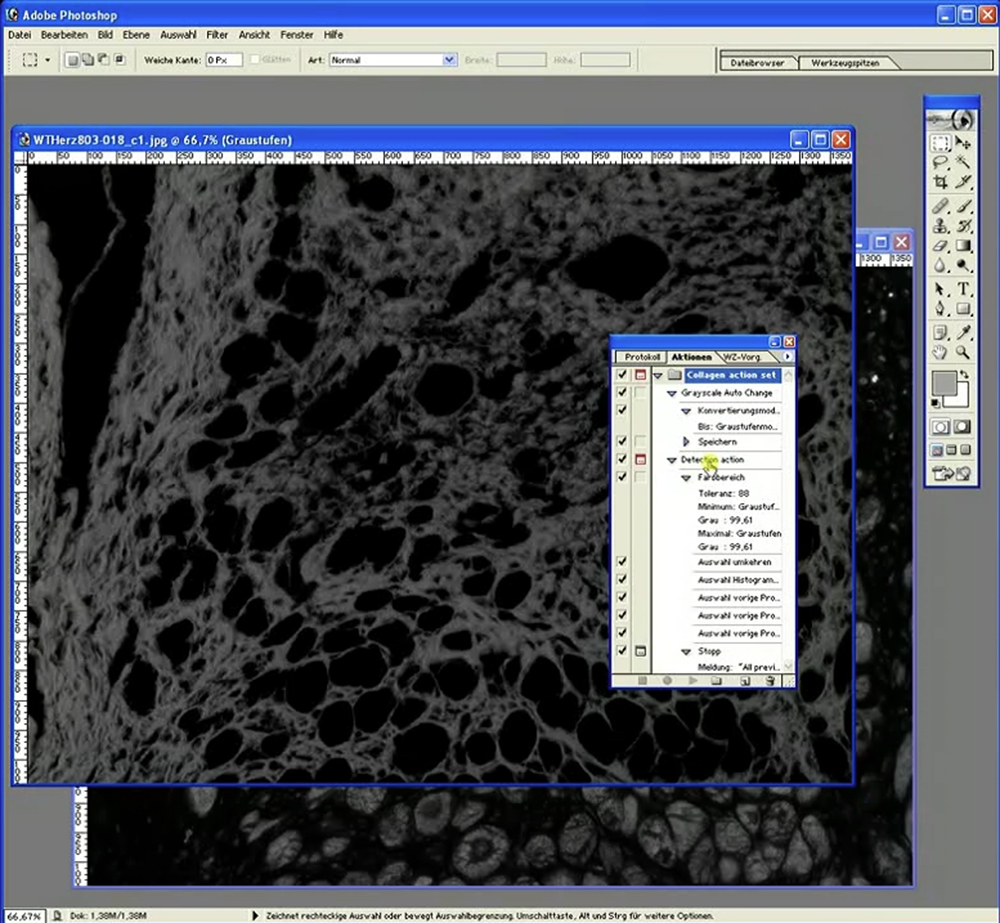

Supplement: Supplementary file 3 [file mmc5.jpg]

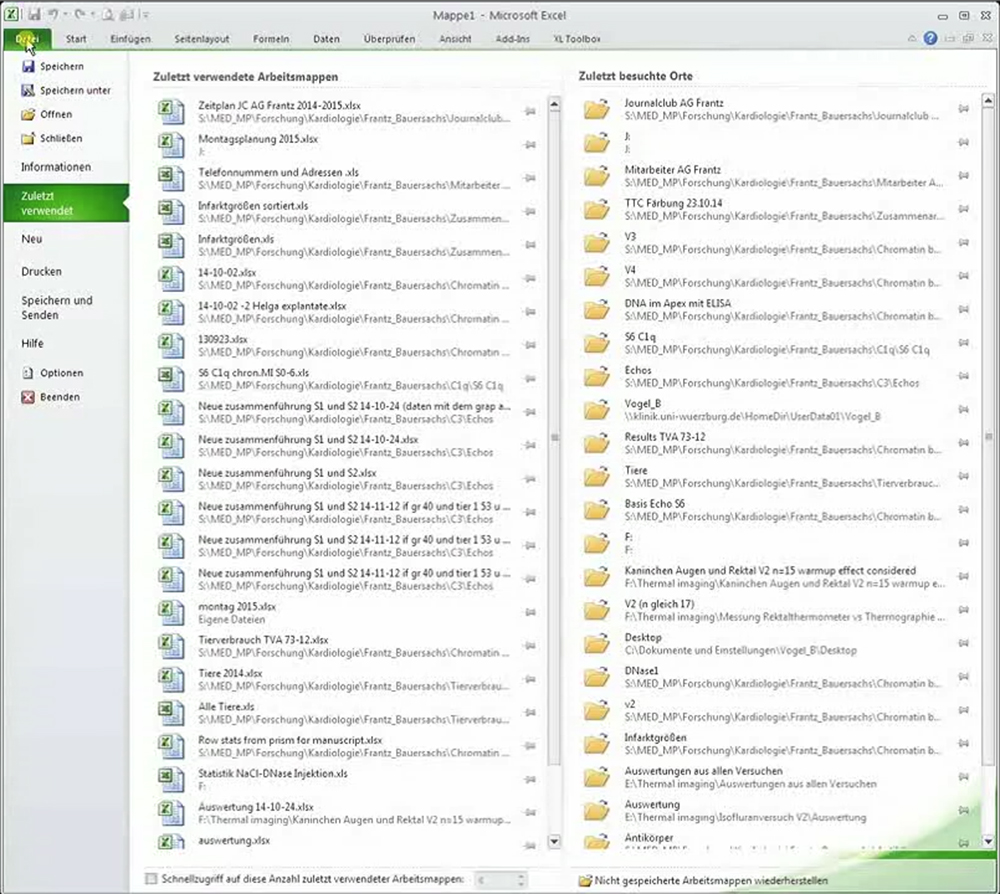

Supplement: Supplementary file 4 [file mmc6.jpg]

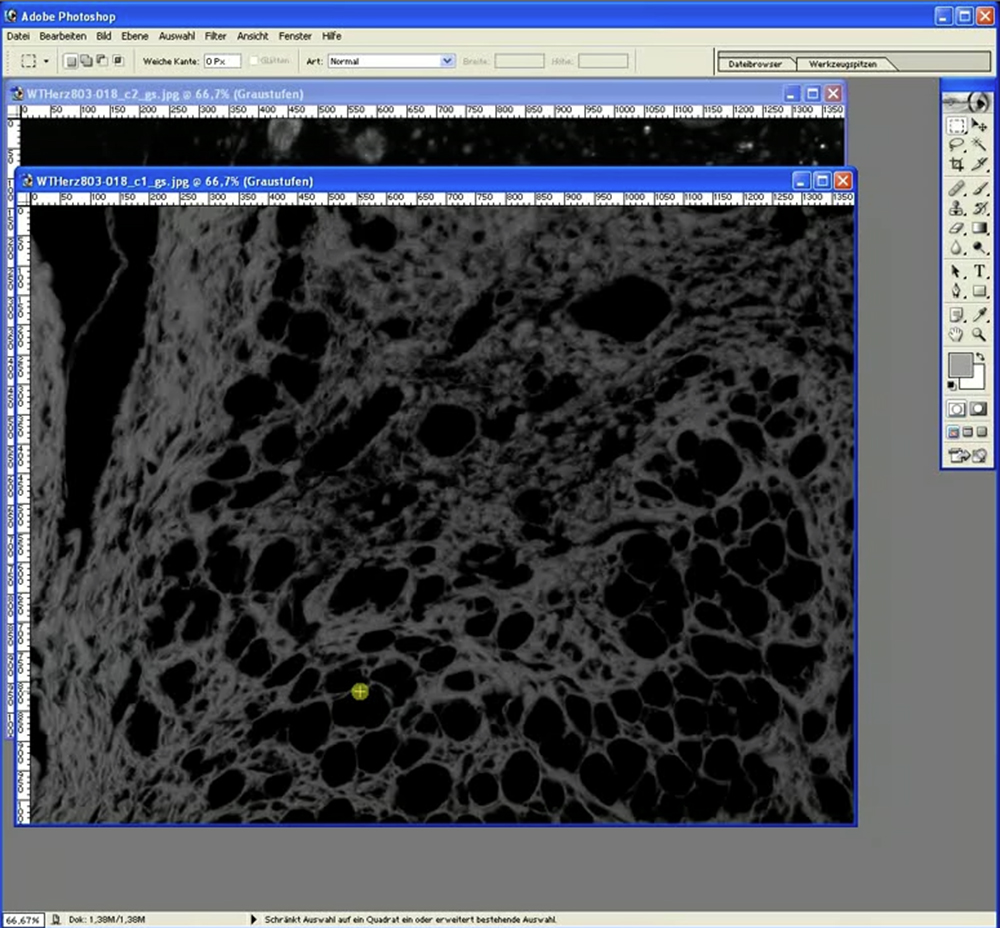

Supplement: Supplementary file 5 [file mmc8.jpg]
